# Luteolin Attenuates IL-1*β*-Induced THP-1 Adhesion to ARPE-19 Cells via Suppression of NF-*κ*B and MAPK Pathways

**DOI:** 10.1155/2020/9421340

**Published:** 2020-10-16

**Authors:** Wen-Chung Huang, Chian-Jiun Liou, Szu-Chuan Shen, Sindy Hu, Chien-Yu Hsiao, Shu-Ju Wu

**Affiliations:** ^1^Graduate Institute of Health Industry Technology, Research Center for Food and Cosmetic Safety, College of Human Ecology, Chang Gung University of Science and Technology, Taoyuan City 33303, Taiwan; ^2^Division of Allergy, Asthma, and Rheumatology, Department of Pediatrics, Chang Gung Memorial Hospital, Linkou, Taoyuan City 33303, Taiwan; ^3^Department of Nursing, Division of Basic Medical Sciences, Research Center for Chinese Herbal Medicine, and Graduate Institute of Health Industry Technology, Chang Gung University of Science and Technology, Taoyuan 33303, Taiwan; ^4^Graduate Program of Nutrition Science, National Taiwan Normal University, 88 Ting-Chow Rd, Sec 4, Taipei, Taiwan; ^5^Department of Cosmetic Science, College of Human Ecology, Chang Gung University of Science and Technology, Guishan Dist., Taoyuan City 33303, Taiwan; ^6^Department of Dermatology, Aesthetic Medical Center, Chang Gung Memorial Hospital, Linkou, Taoyuan City 33303, Taiwan; ^7^Department of Nutrition and Health Sciences, Research Center for Chinese Herbal Medicine, College of Human Ecology, Chang Gung University of Science and Technology, Taoyuan City 33303, Taiwan

## Abstract

Cytokine-induced endothelial dysfunction leads to inflammation and vascular adhesion molecule production in retinal pigment epithelium (RPE) cells. Inflammation is a critical mediator in retinal degeneration (RD) diseases, including age-related macular degeneration (AMD), and RD progression may be prevented through anti-inflammatory activity in RPE cells. The flavonoid polyphenol luteolin (LU) has anti-inflammatory and antidiabetes activities, but its effects regarding retinal protection remain unknown. Here, we examined the ability of luteolin to alleviate markers of inflammation related to RD in cytokine-primed APPE-19 cells. We found that luteolin decreased the levels of interleukin- (IL-) 6, IL-8, soluble intercellular adhesion molecule-1 (sICAM-1), and monocyte chemoattractant protein-1 (MCP-1) and attenuated adherence of the human monocytic leukemia cell line THP-1 to IL-1*β*-stimulated ARPE-19 cells. Luteolin also increased anti-inflammatory protein heme oxygenase-1 (HO-1) levels. Interestingly, luteolin induced protein kinase B (AKT) phosphorylation, thus inhibiting nuclear factor- (NF-) *κ*B transfer from cytoplasm into the nucleus and suppressing mitogen-activated protein kinase (MAPK) inflammatory pathways. Furthermore, cotreatment with MAPK inhibitors and luteolin decreased inflammatory cytokine and chemokine levels, and further suppressed THP-1 adhesion. Overall, these results provide evidence that luteolin protects ARPE-19 cells from IL-1*β*-stimulated increases of IL-6, IL-8, sICAM-1, and MCP-1 production by blocking the activation of MAPK and NF-*κ*B signaling pathways, thus ameliorating the inflammatory response.

## 1. Introduction

The retinal pigment epithelium (RPE) is a single layer of pigment cells, which is in close contact with photoreceptors and maintains visual function [1]. RPE exposure to ultraviolet/visible light leads to oxidative stress and chronic inflammation in the retinal tissue. Inflammation clearly plays a role in the development of age-related macular degeneration (AMD), which is a cause of severe irreversible visual impairment in elderly persons and in diabetic retinopathy [2, 3]. A variety of factors promote retinal tissue degeneration and AMD progression, including genetic and environmental factors, aging, and oxidative stress [4]. AMD involves reduced photoreceptor cells and retinal pigment epithelium dysfunction in the macula and can be classified as “dry AMD” or “wet/exudative/neovascular AMD.” Compared to neovascular AMD, dry AMD has a higher incidence, but involves less vision degradation and is thus less frequently a cause of “blindness” [5, 6]. Neovascular AMD is characterized by excessive choroidal neovascularization (CNV) under the retina, leading to retinal edema and even detachment, thereby causing vision loss. Notably, dry AMD can potentially evolve into neovascular AMD, leading to irreversible vision loss [7, 8]. Therefore, the best means of avoiding vision deterioration in elderly persons and cases of diabetic retinopathy is to prevent macular degeneration.

Available data suggest that a number of inflammatory cytokines and chemokines are elevated in the serum or ocular tissue of AMD patients—including interleukin-6 (IL-6), IL-8, monocyte chemotactic protein (MCP-1), and intercellular adhesion molecule (ICAM-1) [9–11]. Notably, IL-6 levels are increased in the intraocular fluids of patients with neovascular AMD compared to controls, and findings suggest that IL-6 and IL-8 are proangiogenic in AMD progression [12, 13]. Intraocular MCP-1 is a chemokine for monocyte recruitment, and ICAM-1 is an adhesion molecule that facilitates leukocyte transmigration. Studies have reported that elevated MCP-1 levels contribute to subfoveal neovascular membrane development and the degree of macular edema in eyes with exudative AMD and that elevated soluble ICAM-1 (sICAM-1) levels are correlated with choroidal neovascularization [13, 14]. Inflammatory mediators, including chemokines and cytokines, can upregulate ICAM-1 expression. IL-6 could directly or indirectly activate the leukocytes to induce retinal inflammation. It has been found that MCP-1 recruits leukocytes to sites of injury and activates ICAM-1; thus, ICAM-1 induced leukocyte-endothelial interactions and promoted leukocyte migration into extravascular locations involved in the inflammatory response [14, 15]. More studies found that IL-8 and MCP-1 could attract neutrophils and monocytes to migrate into inflammatory tissues. IL-1*β* is a proinflammatory cytokine and promotes the upregulation of chemokines in RPE as model of focal retinal degeneration [15]. In the present study, we evaluated the ability of the luteolin to modulate inflammation in ARPE-19 cells-THP-1 monocytes interactions. The levels of the inflammatory cytokines IL-6, IL-8, MCP-1, and ICAM-1 in ocular tissue are significantly associated with exudative AMD occurrence and progression [4]. Moreover, IL-1*β* activates inflammatory-related pathways, including nuclear factor- (NF-) *κ*B and mitogen-activated protein kinase (MAPK) signaling, thus enhancing the production of the proinflammatory mediators MCP-1, IL-6, cyclooxygenase-2 (COX-2), and inducible nitric oxide synthase (iNOS). Proinflammatory mediators are closely associated with the development and progression of retinal degenerative diseases [15, 16].

Luteolin (Lu), 3',4',5,7-tetrahydroxyflavone, is a flavonoid polyphenolic compound found in numerous vegetables (e.g., celery, carrots, broccoli, and peppers), fruits (e.g., apple, mango, blueberry, peaches, and prunes), and herbs (e.g., chrysanthemum flowers and *Taraxacum mongolicum*) [17–20]. In previous studies, it was demonstrate that luteolin shows anti-inflammatory, anticancer, neuroprotective, and antiviral properties in vitro and in animal models [21–25]. Thus, luteolin has been investigated for potential use in the treatment of obesity [26] and obesity-related diseases and for antidiabetic [27–29] and neuroprotective therapies [30, 31]. In ophthalmological research, luteolin protects against oxidative stress-related damage and decreases inflammation in ARPE-19 cells [32, 33]. Luteolin is protective against diabetes-induced progression of retinopathy and reportedly inhibits expressions of the inflammation-related markers NF-*κ*B and IL-1*β*, decreases levels of the lipid peroxidation product malondialdehyde (MDA), and increases the antioxidant glutathione (GSH) in diabetes-induced oxidative stress in the retina [34]. These findings indicate the potential to use luteolin for prevention and treatment of retinal inflammatory diseases.

**Figure 1:**
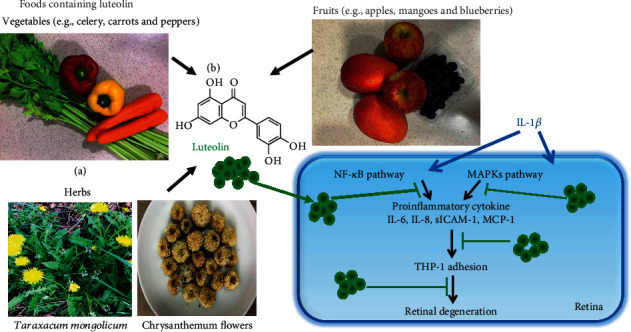
Experimental abstract. (a) Foods containing luteolin. (b) The structure of luteolin. (c) Pathways likely related to the anti-inflammatory activity of luteolin in IL-1*β*-stimulated ARPE-19 cells.

Based on the available data, we speculate that IL-6, IL-8, MCP-1, and sICAM-1 could be target molecules for AMD therapy or prevention. In the present study, we aimed to assess the mechanisms underlying the luteolin-induced anti-inflammatory effects in ARPE-19 cells stimulated by the proinflammatory cytokine IL-1*β* (Figure 1).

## 2. Materials and Methods

### 2.1. Materials

Luteolin (≥98% purity by TLC), cell counting kit-8 assays (CCK-8), and DAPI solution were purchased from Sigma-Aldrich (St. Louis, MO, USA). Human recombinant proinflammatory cytokine IL-1*β* and enzyme-linked immunosorbent assay (ELISA) kits were purchased from R&D Systems (Minneapolis, MN, USA). The inhibitors PD98059, SP600125, SB202190, and Bay 117082 were purchased from Enzo Life Sciences (Farmingdale, NY, USA). Antibodies against *β*-actin, COX-2, iNOS, HO-1, AKT, and phosphorylated- (phospho-) AKT were purchased from Santa Cruz Biotechnology (Santa Cruz, CA, USA). Antibodies against JNK, ERK, p38, phospho-JNK, phospho-ERK, and phospho-p38 were purchased from Millipore (Billerica, MA, USA).

### 2.2. Preparation of Luteolin and Cell Culture

Luteolin was dissolved in dimethyl sulfoxide (DMSO) to prepare a 100 mM stock solution, which was stored at −20°C. The final culture medium had a DMSO concentration of ≤0.1%, as previously described [16]. The ARPE-19 human retinal pigment epithelial cell line was purchased from Bioresource Collection and Research Center (BCRC, Taiwan) and cultured in Dulbecco's modified Eagle's medium (DMEM)/F-12 medium (Invitrogen-Gibco, Paisley, Scotland) containing 10% heat-inactivated fetal bovine serum (FBS; Invitrogen-Gibco) and penicillin G (100 U/mL), streptomycin (100 *μ*g/mL), and gentamycin (50 ng/mL). Cells were subcultured every 2–3 days at 37°C in a humidified atmosphere of 5% CO_2_. ARPE-19 cells (2 × 10^5^) were pretreated with or without various luteolin concentrations (1–30 *μ*M) for 1 h, and then, IL-1*β* (1 *μ*g/mL) was added. After 24 h, the ARPE-19 cells were lysed for western blot analysis, and the media samples were subjected to ELISA analysis. The THP-1 human monocytic leukemia cell line was obtained from American Type Culture Collection (Manassas, VA, USA). THP-1 cells were cultured in RPMI 1640 medium (Gibco) containing FBS (10%) at 37°C in a humidified atmosphere of 5% CO_2_ and subcultured every 3–4 days.

### 2.3. Cell Viability Assay

We assessed the inhibitory effect of luteolin on cell viability using CCK-8 kits (Sigma-Aldrich) as described previously [35]. Cells were seeded at 10^5^ cells/well into 96-well plates and treated with luteolin at concentrations of 1–100 *μ*M for 24 h. After treatment, the CCK-8 solution was added, and the plates were incubated at 37°C for 2 h. Finally, cell viability was measured at 450 nm using a microplate reader (Multiskan FC; Thermo, Waltham, MA, USA). The CCK-8 assay with each concentration was carried out in triplicate, and cell viability was reported as a percentage relative to the cells without luteolin treatment.

### 2.4. ELISA Assay

ARPE-19 cells (10^5^ cells/mL) were pretreated without or with various luteolin concentrations (1–30 *μ*M) in 24-well plates for 1 h. Then IL-1*β* (1 ng/mL) was added, and the cells were cultured for 24 h. Specific ELISA kits were used to measure the levels of IL-6, IL-8 MCP-1, and ICAM-1 in the supernatants, following the manufacturers' instructions. The OD at 450 nm was determined using a microplate reader (Multiskan FC; Thermo).

### 2.5. Preparation of Total Proteins

ARPE-19 cells (8 cells/mL) were pretreated with or without luteolin (1–30 *μ*M) for 1 h. In 6-well plates, the cells were then stimulated with or without IL-1*β* (1 ng/mL) for 24 h to evaluate total protein content, or for 30 min to evaluate phosphorylated protein content. Cells were harvested with 300 mL lysis buffer (50 mM Tris–HCl, pH 7.4; 1 mM EDTA; 150 mM NaCl; 1 mM DTT; 0.5% NP40; and 0.1% sodium dodecyl sulfate (SDS)) containing protease inhibitor cocktail and phosphatase inhibitors (Sigma, St. Louis, MO, USA). The BCA assay kit (Pierce) was used to quantitate all protein concentrations.

### 2.6. Western Blot Analysis

Protein samples were separated on 10% SDS polyacrylamide gels and then transferred to polyvinylidene fluoride (PVDF) membranes (Millipore, Billerica, MA, USA). Next, the PVDF membranes were incubated overnight at 4°C with specific primary antibodies against *β*-actin (Sigma), COX-2, iNOS, HO-1, AKT, pAKT (Santa Cruz, CA, USA), JNK, pJNK, p38, and pp38 (Cell Signaling Technology, Danvers, MA, USA). Then, the membranes were washed three times using tris-buffered saline with Tween 20 (TBST) buffer and incubated with secondary antibodies at room temperature for 1 h. Proteins were detected using Luminol/Enhancer solution (Millipore), and signals were detected using the BioSpectrum 600 system (UVP, Upland, CA, USA) to quantitate protein bands.

### 2.7. Monocyte Adhesion Assay

In the first step, AREP19 cells (2 × 10^5^ cells/mL) were pretreated with luteolin (1, 3, 10, and 30 *μ*M) or inhibitors (10 *μ*M SP600125, 10 *μ*M PD98059, 10 *μ*M SB202190, or 5 *μ*M Bay 11-7082) for 1 h in DMEM medium. THP-1 cells (5 × 10^5^ cells/mL) were labeled with fluorescent dye (5 *μ*M calcein-AM) in RPMI-1640 medium at 37°C for 30 min in the dark and then washed by centrifugation. Second, the labeled THP-1 cells were cocultured with the ARPE-19 cells in plates for 1 h, and then, the cells were washed three times with PBS to remove nonadherent THP-1 cells. Finally, the extent of adhesion of THP-1 cells to ARPE19 cells was observed under fluorescence microscope (4 per view; magnification, ×200; Olympus Corporation, Tokyo, Japan). The control groups were treated with IL-1*β* alone, and all experiments were repeated three times.

### 2.8. Immunofluorescence Staining

ARPE19 cells were seeded into 6-well plates until reaching 50–60% confluence and pretreated with or without luteolin (1, 3, 10, and 30 *μ*M) for 1 h, followed by addition of IL-1*β* for 15 min. Then, the medium was suctioned out, and the cells were washed with PBS. The cells were fixed with 4% (*w*/*v*) paraformaldehyde and incubated with anti-NF-*κ*B p65 antibody overnight at 4°C. The next day, the medium was removed, and the cells were washed with PBS and then incubated with secondary antibodies at room temperature for 1 h. Then, the cells were washed 2–3 times with PBS, followed by the addition of fluorescent dye (BODIPY493/503 or BODIPY581/591). The cells were again washed with PBS to remove the dye, and 4',6-diamidino-2-phenylindole (DAPI; Sigma) was added to stain the nucleus. Finally, images were acquired using a fluorescence microscope (Olympus, Tokyo, Japan).

### 2.9. Statistical Analysis

Image Lab software (Bio-Rad) was used to quantify the intensity of western blot bands, and ImageJ software (W. Rasband, NIH, USA) to determine the numbers of THP-1 cells in the adhesion assay. Data were presented as the mean ± standard deviation (SD) from at least three independent experiments. Statistical analyses included one-way analysis of variance (ANOVA) followed by the Tukey's post hoc test. A *p* value of < 0.05 was considered significant.

## 3. Results

### 3.1. Luteolin Inhibited Inflammatory Mediator Expression and Increased Anti-Inflammatory Protein HO-1 Expression in IL-1*β*-Stimulated ARPE19 Cells

**Figure 2:**
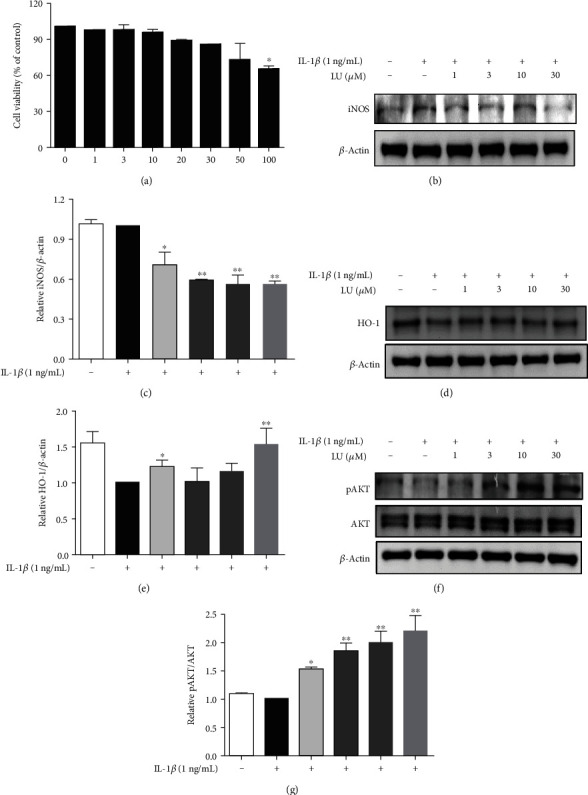
Luteolin inhibited inflammatory mediator expression and increased anti-inflammatory protein HO-1 expression in IL-1*β*-stimulated ARPE19 cells. (a) Cell viability of ARPE19 cells treated with various luteolin (LU) concentrations (0–100 *μ*M). Cells were pretreated with luteolin for 1 h, then stimulated with IL-1*β* (1 ng/mL) for 24 h. (b) Cells were pretreated with different LU doses and then incubated with IL-1*β* (1 ng/mL) for 30 min or 24 h. Western blots show iNOS protein expression. (c) The fold-change in iNOS protein expression was measured relative to *β*-actin expression. (d) Western blots show HO-1 protein expression. (e) The fold-change in HO-1 protein expression was measured relative to *β*-actin expression. (f) Western blots show phosphorylated AKT protein expression. (g) The fold-change in pAKT protein expression was measured relative to AKT expression. Data represent the mean ± SD. ∗*p* < 0.05, ∗∗*p* < 0.01, compared to ARPE-19 cells stimulated with IL-1*β* alone.

First, we performed a CCK-8 assay to assess luteolin cytotoxicity in ARPE-19 cells. Luteolin concentrations of ≤50 *μ*M showed no significant cytotoxicity in ARPE-19 cells, while cell numbers were significantly reduced at concentrations of ≥100 *μ*M (Figure 2(a)). Therefore, all subsequent experiments were performed using 1–30 *μ*M luteolin. ARPE-19 cells were seeded in 6-well plates, treated with luteolin, and then stimulated with 1 ng/mL IL-1*β*. Compared with IL-1*β* alone, additional treatment with luteolin at ≥1 *μ*M significantly inhibited expression of the inflammatory mediator iNOS (Figures 2(b) and 2(c)) but did not significantly inhibit COX-2 expression (data not shown). Notably, compared with IL-1*β* alone, treatment with 1 and 30 *μ*M luteolin significantly increased expression of the anti-inflammatory protein HO-1 (Figures 2(d) and 2(e)). Previous studies have indicated that AKT activation may contribute to inhibiting the NF-*κ*B inflammatory pathway in inflammation-related diseases [36]. Our results showed that luteolin at ≥1 *μ*M enhanced the expression of phosphorylated AKT proteins in IL-1*β*-stimulated ARPE19 cells compared with in cells treated with IL-1*β* alone (Figures 2(f) and 2(g)).

### 3.2. Luteolin Inhibited Inflammation-Related Cytokines and Attenuated THP-1 Cell Adherence to IL-1*β*-Stimulated ARPE-19 Cells

**Figure 3:**
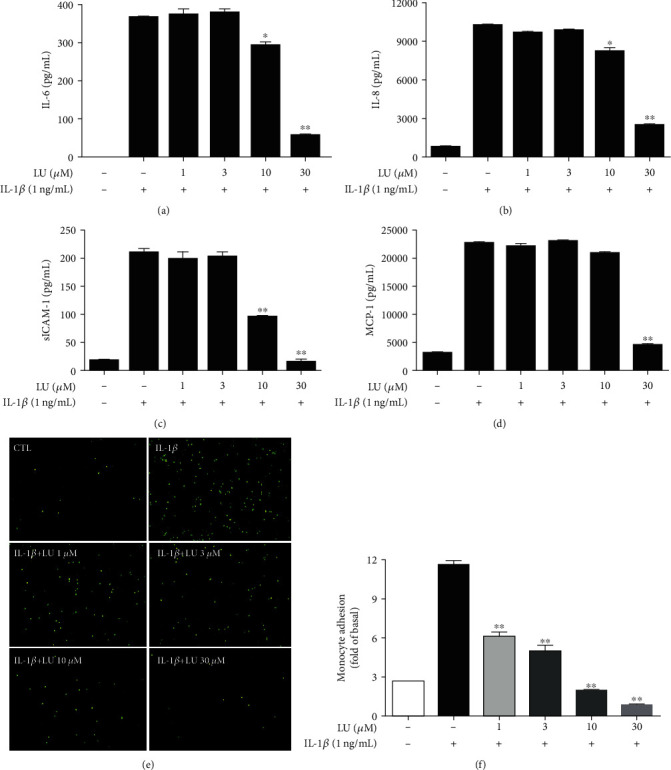
Luteolin inhibited inflammation-related cytokine expression and attenuated THP-1 cell adherence to IL-1*β*-stimulated ARPE-19 cells. Cells were pretreated with different doses of luteolin (LU) and then incubated with IL-1*β* (1 ng/mL) for 24 h. ELISA results showed the levels of (a) IL-6, (b) IL-8, (c) sICAM-1, and (d) MCP-1. (e) LU significantly suppressed THP-1 cell adherence to IL-1*β*-stimulated ARPE-19 cells. Fluorescent-labeled THP-1 cells (green) were cocultured with control (CTL) or IL-1*β*-stimulated ARPE-19 cells in the absence or presence of the indicated LU concentrations. (f) The fluorescence intensity revealed THP-1 cell adhesion to IL-1*β*-stimulated ARPE-19 cells, which was used to quantify calcein-AM fluorescence. The data represent the mean ± SD. ∗*p* < 0.05, ∗∗*p* < 0.01, compared to ARPE-19 cells stimulated with IL-1*β* alone.

ARPE-19 cells were pretreated with luteolin (1, 3, 10, or 30 *μ*M) for 1 h, and then, 1 ng/mL IL-1*β* was added for 24 h. IL-1*β* treatment alone significantly stimulated ARPE19 cells to release cytokines and chemokines compared with control cells. Luteolin at concentrations of 10 and 30 *μ*M significantly inhibited the IL-1*β*-induced release of IL-6 and IL-8 and decreased the levels of the cell adhesion molecule sICAM-1. Additionally, treatment with 30 *μ*M luteolin significantly decreased MCP-1 levels compared to treatment with IL-1*β* alone (Figures 3(a)–3(d)). Since luteolin concentrations of ≥10 *μ*M strongly inhibited sICAM-1 levels, we further investigated whether luteolin attenuated THP-1 cell adhesion to IL-1*β*-stimulated ARPE-19 cells. Pretreatment with luteolin significantly inhibited THP-1 cell adherence to IL-1*β*-stimulated ARPE-19 cells, compared to samples treated with IL-1*β* alone (Figures 3(e) and 3(f)).

### 3.3. Luteolin Inhibited NF-*κ*B Activation to Suppress THP-1 Cell Adherence to IL-1*β*-Stimulated ARPE-19 Cells

**Figure 4:**
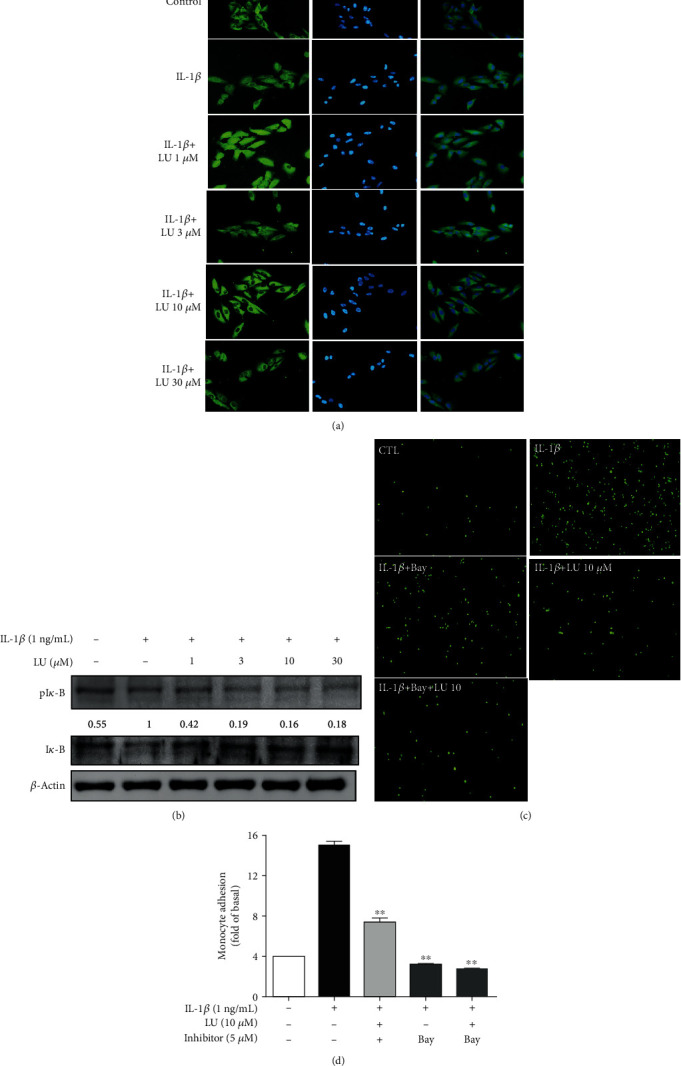
Luteolin inhibited NF-*κ*B activation and thereby suppressed THP-1 cell adherence to IL-1*β*-stimulated ARPE-19 cells. Cells were pretreated with different doses of luteolin (LU) and then incubated with IL-1*β* (1 ng/mL) for 15 min. NF-*κ*B p65 translocation was evaluated by immunofluorescence staining. Green: location of the p65 subunit. Blue: DAPI for nuclear staining. (a) DAPI staining revealed that luteolin attenuated NF-*κ*B p65 translocation in IL-1*β*-stimulated ARPE-19 cells. (b) Western blots show the levels of phosphorylated I*κ*B protein expression. (c) Luteolin suppressed THP-1 adhesion. ARPE-19 cells were pretreated with 10 *μ*M luteolin or 5 *μ*M Bay 117082 for 1 h and then cocultured with labeled THP-1 cells. (d) Fluorescence intensity showed THP-1 cell adhesion to IL-1*β*-stimulated ARPE-19 cells, which was used to quantify calcein-AM fluorescence. Data represent the mean ± SD. ∗∗*p* < 0.01, compared to ARPE-19 cells stimulated with IL-1*β* alone.

We observed that luteolin significantly increased AKT activation (Figures 2(f) and 2(g)), and pAKT has previously been associated with inhibition of the NF-*κ*B inflammatory pathway [37]. Therefore, we evaluated whether luteolin inhibited NF-*κ*B in relation to the suppression of THP-1 cell adherence to IL-1*β*-stimulated ARPE-19 cells. Cells were pretreated with or without luteolin (1–30 *μ*M) for 1 h, then stimulation with IL-1*β* (1 ng/mL) for 15 min, to investigate whether luteolin inhibited NF-*κ*B p65 translocation. Immunofluorescence staining revealed that luteolin at ≥10 *μ*M suppressed NF-*κ*B p65 translocation from the cytoplasm into the nucleus and that the p65 subunit was retained in the cytoplasm in IL-*β*-activated ARPE-19 cells (Figure 4(a)). Then, we evaluated whether luteolin inhibited I*κ*B phosphorylation. Treatment with luteolin at ≥1 *μ*M significantly inhibited phosphorylated I*κ*B expression, compared with IL-1*β* alone (Figure 4(b)). We further investigated whether luteolin decreased THP-1 cell adherence to ARPE-19 cells via inhibition of NF-*κ*B p65 activation. First, ARPE-19 cells were pretreated with either luteolin (10 *μ*M) or Bay 11-7082 (5 *μ*M) for 1 h and then stimulated with IL-1*β*. Second, these pretreated ARPE-19 cells were cocultured with labeled THP-1 cells. Our results showed that both luteolin and Bay 11-7082 pretreatment significantly inhibited THP-1 cell adherence to IL-1*β*-stimulated ARPE-19 cells compared to cells treated with IL-1*β* alone (Figures 4(b) and 4(c)).

### 3.4. Luteolin Blocked MAPK Inflammatory Pathways and MAPK Inhibitors Decreased THP-1 Cell Adherence to IL-1*β*-Stimulated ARPE-19 Cells

**Figure 5:**
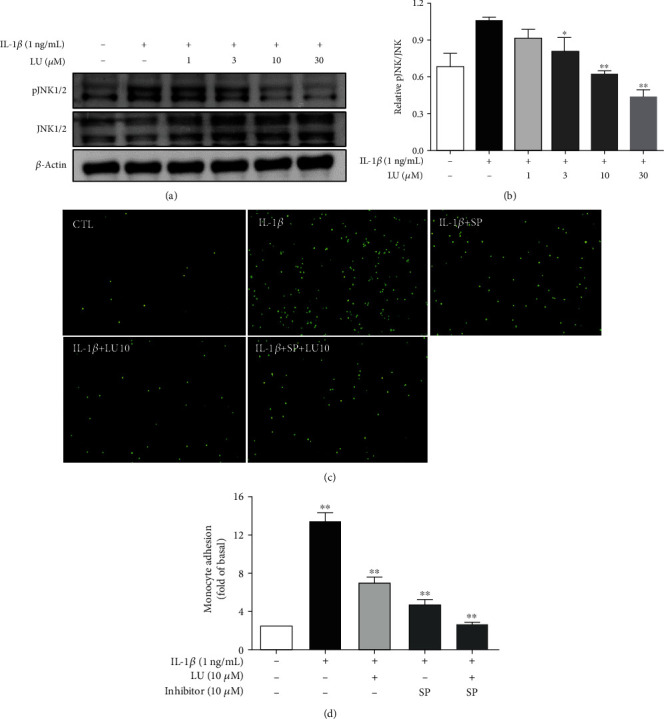
Luteolin blocked JNK phosphorylation, and the JNK inhibitor SP60012 (SP) decreased THP-1 cell adherence to IL-1*β*-stimulated ARPE-19 cells. (a) Western blots show the levels of phosphorylated JNK protein expression. (b) The fold-change in pJNK protein expression was measured relative to JNK expression. (c) ARPE-19 cells were pretreated with 10 *μ*M luteolin or JNK inhibitor (SP600125) for 1 h and then cocultured with labeled THP-1 cells. (d) The fluorescence intensity was used to quantify calcein-AM fluorescence. Data represent the mean ± SD. ∗*p* < 0.05, ∗∗*p* < 0.01, compared to ARPE-19 cells stimulated with IL-1*β* alone.

**Figure 6:**
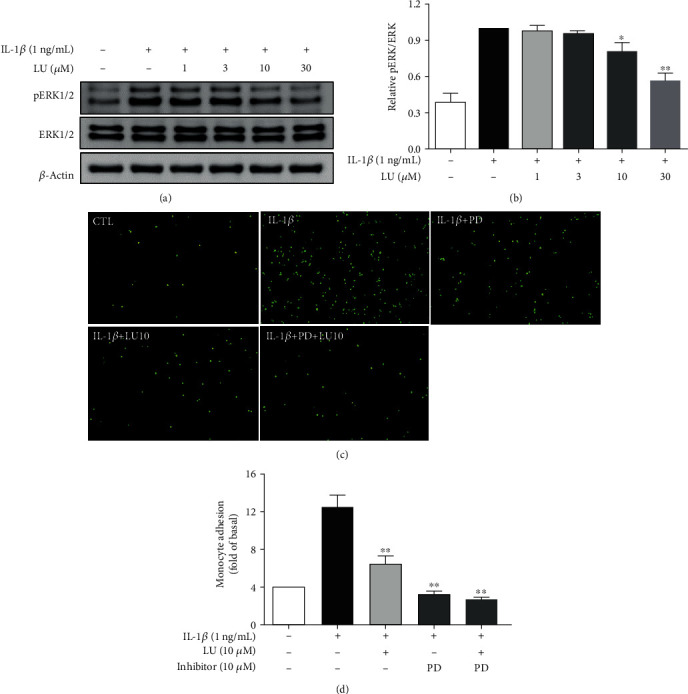
Luteolin blocked ERK phosphorylation and the ERK inhibitor PD98059 (PD) decreased THP-1 cell adherence to IL-1*β*-stimulated ARPE-19 cells. (a) Western blots show levels of phosphorylated ERK protein expression. (b) The fold-change in pERK protein expression was measured relative to ERK expression. (c) ARPE-19 cells were pretreated with 10 *μ*M luteolin or ERK inhibitor (PD98059) for 1 h and then cocultured with labeled THP-1 cells. (d) The fluorescence intensity was used to quantify calcein-AM fluorescence. Data represent the mean ± SD. ∗*p* < 0.05, ∗∗*p* < 0.01, compared to ARPE-19 cells stimulated with IL-1*β* alone.

**Figure 7:**
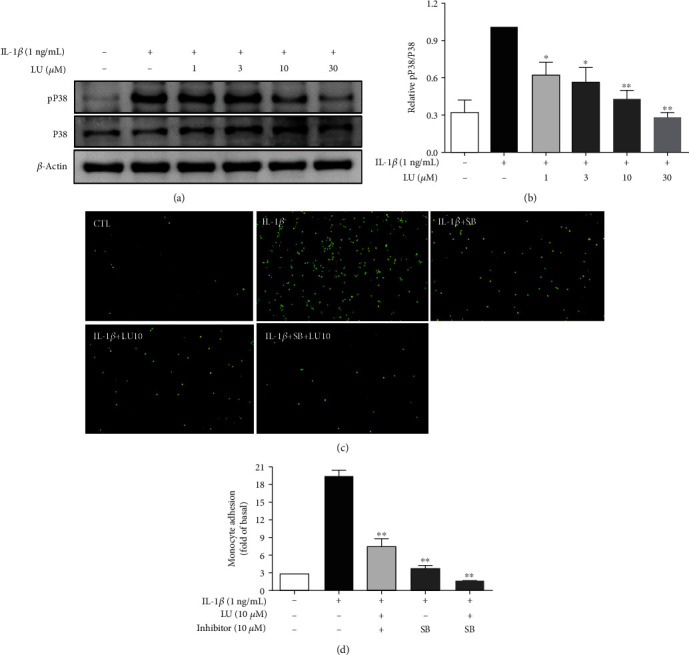
Luteolin blocked P38 phosphorylation, and the P38 inhibitor SB202190 (SB) decreased THP-1 cell adherence to IL-1*β*-stimulated ARPE-19 cells. (a) Western blots show levels of phosphorylated P38 protein expression. (b) The fold-change in pP38 protein expression was measured relative to P38 expression. (c) ARPE-19 cells were pretreated with 10 *μ*M luteolin or P38 inhibitor (SB203580) for 1 h and then cocultured with labeled THP-1 cells. (d) The fluorescence intensity was used to quantify calcein-AM fluorescence. Data represent the mean ± SD. ∗*p* < 0.05, ∗∗*p* < 0.01, compared to ARPE-19 cells stimulated with IL-1*β* alone.

We next evaluated whether luteolin inhibited MAPK phosphorylation and if this was related to the decreased THP-1 cell adherence to IL-1*β*-stimulated ARPE-19 cells. First, cells were pretreated with luteolin (1–30 *μ*M) for 1 h and then incubated with IL-1*β* (1 ng/mL) for 30 min or 24 h to evaluate the expression of MAPK signaling proteins. Our results showed that luteolin at ≥3 *μ*M significantly decreased phosphorylated c-JUN N-terminal kinase (pJNK) 1/2 expression, luteolin at ≥10 *μ*M significantly decreased phosphorylated extracellular signal-regulated kinase (pERK) 1/2 expression, and luteolin at ≥3 *μ*M significantly decreased phosphorylated p38 protein expression (Figures 5(a), 5(b), 6(a), 6(b), 7(a), and 7(b)). We further evaluated whether the MAPK-inhibiting effects of luteolin were associated with decreased THP-1 adherence to ARPE-19 cells. ARPE-19 cells were pretreated with 10 *μ*M luteolin and/or 10 *μ*M of the JNK inhibitor SP60012, the ERK1/2 inhibitor PD98059, or the p38 inhibitor SB202190 for 1 h, followed by incubation with IL-1*β* (1 ng/mL) for 24 h. All tested pretreatments decreased THP cell adherence to ARPE-19 cells. Moreover, combined pretreatment with luteolin plus SP60012 or luteolin plus SB202190 resulted in significantly greater reductions of THP-1 adhesion compared to treatment with any of these agents alone (Figures 5(c), 5(d), 6(c), 6(d), 7(c), and 7(d)).

### 3.5. MAPK Inhibitors Mediated the Levels of Cytokines and Chemokines in IL-1*β*-Stimulated ARPE-19 Cells

**Figure 8:**
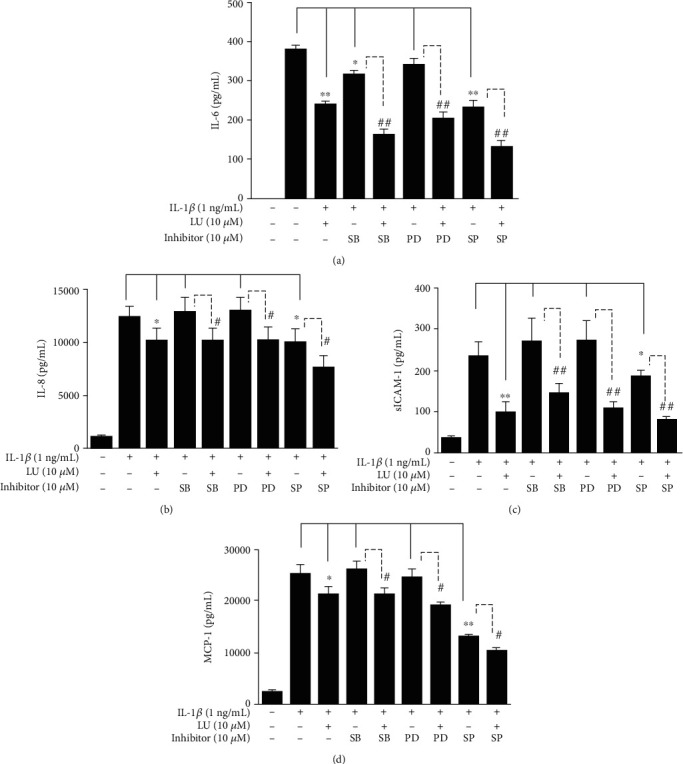
MAPK inhibitors mediated the expression levels of cytokines and chemokines in IL-1*β*-stimulated ARPE-19 cells. ARPE-19 cells were treated with MAPK inhibitors (10 *μ*M SB203580 (SB), 10 *μ*M PD98059 (PD), or 10 *μ*M SP600125 (SP)) with or without 10 *μ*M luteolin and then incubated with IL-1*β* for 24 h. ELISA results showed the levels of (a) IL-6, (b) IL-8, (c) sICAM-1, and (d) MCP-1. Data represent the mean ± SD. ∗*p* < 0.05, ∗∗*p* < 0.01, compared to ARPE-19 cells stimulated with IL-1*β* alone. ^#^*p* < 0.05, ^##^*p* < 0.01, compared to IL-1*β*-stimulated ARPE-19 cells pretreated with only MAPK inhibitor.

We observed that luteolin significantly decreased the release of IL-6, IL-8, sICAM-1, and MCP-1 in IL-1*β*-stimulated ARPE-19 cells (Figures 3(a)–3(d)), as well as significantly suppressed MAPK pathways (Figures 5(a), 5(b), 6(a), 6(b), 7(a), and 7(b)). Next, we investigated whether MAPK inhibitors could attenuate the IL-1*β*-stimulated production of the inflammatory cytokines IL-6, IL-8, and MCP-1. ARPE-19 cells were pretreated with luteolin and/or MAPK inhibitors (10 *μ*M SB202190, 10 *μ*M PD98059, or 10 *μ*M SP600125) for 1 h and then incubated with 1 ng/mL IL-1*β* for 24 h. Interestingly, the pretreatment with MAPK inhibitors and luteolin significantly reduced the levels of IL-6, IL-8, sICAM-1, and MCP-1 in IL-1*β*-stimulated ARPE-19 cells (Figures 8(a)–8(d)). These results suggested that in IL-1*β*-stimulated ARPE-19 cells, luteolin suppressed the expression of IL-6, IL-8, sICAM-1, and MCP-1 by influencing the phosphorylation of p38, ERK1/2, and JNK1/2.

## 4. Discussion

In many retinal degenerative (RD) diseases, the pathogenesis is inflammation-induced, involving the recruitment and activation of microglia and macrophages, the expression of inflammatory mediators (COX-2 and iNOS), and photoreceptor cell death. The proinflammatory cytokine IL-1*β* triggers inflammatory responses and attracts inflammatory cells to migrate into the retina, promoting retinal impairment and degeneration in RD diseases [38, 39]. Numerous studies reported that IL-1*β* activates the expression of other proinflammatory cytokines and modulates chemokine expression. Proinflammatory cytokines can target and induce retinal inflammation in RD pathogenesis [36, 37] and the phytochemical luteolin has excellent anti-inflammatory properties [32, 33]. Therefore, here we performed a detailed exploration of the anti-inflammatory effects of luteolin in IL-1*β*-stimulated ARPE19 cells.

Prior studies have indicated that the cytokines IL-6 and IL-8 are proangiogenic, while the chemokine MCP-1 and cell adhesion molecule ICAM-1 facilitate leukocyte transmigration into ocular tissue, in AMD development and progression [4, 12–14]. Elevated IL-1*β* levels in the vitreous or retina lead to photoreceptor cell death in retinal detachment patients and in a mouse model, while reduced IL-1*β* levels inhibit photoreceptor cell death [40]. In the present study, we demonstrated that luteolin significant inhibited cytokine and chemokine release in IL-1*β*-stimulated ARPE-19 cells (Figures 3(a)–3(d)). Prior studies have also shown that ICAM-1 is upregulated in response to inflammatory mediators and mediates leukocyte adhesion and transmigration on the RPE, while decreased ICAM-1 levels can suppress monocyte adhesion in RPE cells [41, 42]. Here, we demonstrated that luteolin inhibited sICAM-1 levels and attenuated THP-1 cell adhesion to IL-1*β*-stimulated ARPE-19 cells (Figure 3(e)). We also found that luteolin significantly inhibited iNOS protein expression and increased HO-1 protein expression (Figures 2(b) and 2(d)). These findings support that luteolin is indeed an anti-inflammatory phytochemical that can attenuate proinflammatory cytokine-induced inflammation in ARPE-19 cells.

IL-1*β* activation of NF-*κ*B signaling is closely associated with RD diseases [15, 16] and pAKT is related to inhibition of the NF-*κ*B inflammatory pathway [36]. Previous studies show that IL-1*β*-activates NF-*κ*B, resulting in its translocation from the cytoplasm into the nucleus, followed by induction of cytokine and chemokine expressions in ARPE-19 cells [43]. Our present results showed that luteolin promoted AKT phosphorylation (Figures 2(f) and 2(g)), inhibited NF-*κ*B p65 activation, and suppressed THP-1 cell adhesion (Figure 4). These findings suggest that luteolin promotes AKT phosphorylation to block NF-*κ*B p65 activation and thereby suppresses THP-1 cell adherence to IL-1*β*-stimulated ARPE-19 cells. Many studies have indicated that MAPK signaling plays an important role in AMD [44]. We observed that luteolin decreased the expression levels of IL-6, IL-8, sICAM-1, and MCP-1 in IL-1*β*-stimulated ARPE-19 cells. Thus, we further investigated whether luteolin attenuated inflammation by suppressing MAPK pathways. Our results demonstrated that luteolin significantly inhibited the phosphorylation of the MAPKs JNK 1/2, ERK 1/2, and p38, supporting that luteolin may block MAPK pathways to decrease production of IL-6, IL-8I, sICAM-1, and MCP-1 (Figures 5(a), 5(b), 6(a), 6(b), 7(a), and 7(b)).

Human and animal studies have demonstrated that specific MAPK inhibitors may be potential therapeutic targets for RD disease treatment [44]. To explore the importance of individual MAPKs, we used the MAPK inhibitors SP600125 (JNK 1/2 inhibitor), PD98059 (ERK 1/2 inhibitor), and SB202190 (P38), individually and as cotreatments with luteolin in IL-1*β*-stimulated ARPE-19 cells. We found that luteolin and MAPK inhibitors decreased THP-1 cell adherence to IL-1*β*-stimulated ARPE-19 cells (Figures 5(c), 5(d), 6(c), 6(d), 7(c), and 7(d)). We further found that these MAPK inhibitors attenuated the IL-1*β*-stimulated production of the inflammatory cytokines IL-6, IL-8, sICAM-1, and MCP-1 (Figures 8(a)–8(d)). These results suggested that luteolin blocked MAPK pathways and inhibited the expression of inflammation-related cytokines—thereby suppressing THP-1 adhesion.

## 5. Conclusions

**Figure 9:**
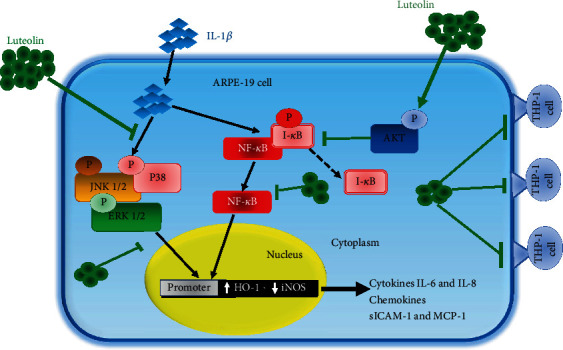
Model explaining the mechanisms via which luteolin inhibits IL-1*β*-induced inflammation in ARPE-19 cells. Luteolin promotes AKT phosphorylation, thus mediating the NF-*κ*B and MAPK pathways, leading to decreased release of inflammatory-related cytokines, and thereby suppressing THP-1 cell adhesion to IL-1*β*-stimulated ARPE-19 cells. Luteolin also increases expression of the anti-inflammatory protein HO-1 and inhibits iNOS protein expression.

Our present data demonstrate that luteolin suppressed proinflammatory cytokine-induced retinal pigment epithelium (RPE) inflammation via inactivation of the NF-*κ*B pathway in IL-1*β*-stimulated ARPE-19 cells. Moreover, cotreatment with MAPK inhibitors plus luteolin attenuated THP-1 cell adhesion to IL-1*β*-stimulated ARPE-19 cells. Importantly, luteolin significantly reduced the expression levels of IL-6, IL-8, sICAM-1, and MCP-1 in IL-1*β*-stimulated ARPE-19 cells. Taken together, our findings suggest that luteolin blocks MAPK pathways, thus decreasing the expression levels of IL-6, IL-8, sICAM-1, and MCP-1, and thereby suppressing THP-1 cell adhesion to IL-1*β*-stimulated ARPE-19 cells (Figure 9). We conclude that the natural agent luteolin may ameliorate inflammation-induced retinal degeneration-related disorders via inhibition of the NF-*κ*B and MAPK pathways in IL-1*β*-stimulated ARPE19 cells.

## Data Availability

The data used to support the findings of this study are available from the corresponding author upon request.

## Abbreviations

RPE: Retinal pigment epithelium cells
RD: Retinal degeneration
AMD: Age-related macular degeneration
ARPE-19 cells: Human retinal pigment epithelial cells
LU: Luteolin
IL-1*β*: Interleukin-1*β*
IL-6: Interleukin-6
IL-8: Interleukin-8
sICAM-1: Soluble intercellular adhesion molecule-1
MCP-1: Monocyte chemoattractant protein-1
HO-1: Heme oxygenase-1
AKT: Protein kinase B
iNOS: Inducible nitric oxide synthase
NF-*κ*B: Nuclear factor-*κ*B
MAPK: Mitogen-activated protein kinase
ELISA: Enzyme-linked immunosorbent assay
JNK: c-JUN N-terminal kinase
ERK: Extracellular signal-regulated kinase.
